# Efficacy of antibiotic and iodoform pastes in non-instrumental endodontic treatment of anterior primary teeth—Protocol for a randomized controlled clinical

**DOI:** 10.1371/journal.pone.0291133

**Published:** 2023-09-08

**Authors:** Ana Paula Taboada Sobral, Elaine Marcilio Santos, Marcela Leticia Leal Gonçalves, Elza Padilha Ferri, Willians Santos Baracho Junior, Thais Gimenez, Juliana Maria Altavista Sagretti Gallo, Anna Carolina Ratto Tempestini Horliana, Lara Jansiski Motta, Sandra Kalil Bussadori

**Affiliations:** 1 Postgraduation Program in Health and Environment, Universidade Metropolitana de Santos, Santos, Brazil; 2 School of Dentistry, Universidade Metropolitana de Santos, Santos, Brazil; 3 Postgraduation Program in Veterinary Medicine in the Coastal Environment, Universidade Metropolitana de Santos, Santos, Brazil; 4 Postgraduation Program in Biophotonics Applied to Health Sciences, Universidade Nove de Julho, São Paulo, Brazil; Universidade Federal de Pelotas, BRAZIL

## Abstract

The maintenance of the deciduous tooth until its physiological exfoliation occurs is one of the main objectives of pediatric dentistry. Endodontic treatment in deciduous teeth resulting from carious or traumatic lesions with pulpal involvement is often necessary and we often find it difficult to perform it, due to the difficult control of the child, the internal anatomy of the root canals, and root resorptions. The non-instrumental endodontic treatment technique (NIET) associated with antimicrobial drugs has advantages such as shorter chair time and less complexity than the conventional technique in which root canal instrumentation is performed. The aim of this study is to carry out a controlled and randomized clinical trial to compare the effectiveness of (NIET) in primary teeth associated with the use of two obturator pastes. One hundred and twenty necrotic deciduous teeth of children aged between 3 and 6 years will be selected; and the teeth will be divided into two groups. In Group 1 and Group 2, root canals will not be instrumented, just irrigated and filled with the respective pastes, antibiotic (CTZ) and iodoform (Guedes-Pinto). Presence of fistula and mobility will be clinically evaluated. The evaluations will be carried out in both groups on the day of treatment and in periods of 1, 3 and 6 months after treatment. For the main outcome, the tooth will be the unit of analysis and the Kaplan-Meier test will be performed to estimate the survival rates of the included teeth. For comparison between the two groups, Student’s t test or Mann-Whitney test will be performed, depending on the normality of the data. In addition, Poisson regression analyzes will be carried out, in order to allow the evaluation of the influence of some variables on the results. For all analyses, the significance value will be adjusted to 5%.

**Trial registration**: NCT04587089 in ClinicalTrials.gov. Approval date: May 15, 2023.

## Introduction

Studies report a reduction in the occurrence of dental caries in the primary dentition throughout the world. However, the prevalence of caries remains high in some population group and childhood cares remains quite frequent. This condition can cause severe destruction and quickly reach the dental pulp [1].

The main causes of pulp inflammation and necrosis in primary teeth are carious lesions and traumatic dental injuries [1, 2]. The occurrence of caries in the primary dentition is quite significant and approximately 75% of teeth with deep caries have pulp involvement [3, 4]. Once the irreversibility of pulp inflammation or tissue necrosis is established, root canal treatment must be performed [2]. There are two treatment options for primary teeth with infected or necrotic pulp: extraction or pulpectomy [3].

Pulpectomy consists of the complete removal of necrotic cells and irreversible infected pulp from an affected tooth so that an asymptomatic, functional tooth remains in the oral cavity until physiological exfoliation [4, 5]. The premature loss of a primary tooth can cause affect the guiding of the eruption of the permanent successor, which can lead to speech problems and harmful oral habits, such as the interposition of the tongue and esthetic consequences [3, 6].

Pulp therapy for primary teeth constitutes complex treatment due to the need for instrumentation, the complexity of the apical delta, the biological cycle of primary teeth, physiological root resorption and rhizolysis as well as long treatment sessions, during which the children are not always cooperative [3, 6, 7].

The most widely used method for the endodontic treatment of primary teeth is performed with manual files and disinfecting irrigating solutions. However, mechanical instrumentation combined with chemical irrigation does not completely eliminate microorganisms from the root canal [8–10]. The aim of endodontic treatment is the maximum disinfection of the root canal system and the prevention of reinfection [11, 12].

The technique called Lesion Sterilization and Tissue Repair Therapy (LSTR) was proposed for non-vital deciduous teeth, with advanced root resorption, strategically important teeth, teeth with bone loss, mobility, radiolucency in the furcation area, uncooperative patients and those who cannot undergo extraction at that time. This technique, however, is contraindicated for patients allergic to any of the components of the antibiotic agents used, extensive internal or external resorption, teeth close to exfoliation, perforation of the pulp floor and children with bacterial endocarditis [13]. The combination of drugs is used to minimize the amount of microorganisms present in the lesion or in the root canal and tissue repair is expected if the lesion is disinfected [14, 15]. When applied for the treatment of necrotic pulps in deciduous teeth, it is performed without instrumenting the root canals, that is, without conventional chemical-mechanical preparation, and an association of antimicrobials is deposited in the entrances of the root canals. The LSTR approach or non-instrumental endodontic treatment (NIET) therefore involves the use of an association of antimicrobial drugs and has advantages such as less time, less complexity, prevention of irritation of the periapical tissues and the germ of the permanent successor, in addition to being able to be used in deciduous teeth that present a rhizolysis process of up to more than a third of the roots [16, 17].

The filling materials for deciduous teeth must have the following properties: resorbable, radiopaque, bactericidal, promote adequate filling and adherence to the walls of the root canals, easily removed when necessary; in addition to not causing damage to the periapical tissues and to the permanent tooth germ, nor altering the color of dental structures. However, there is no single material that fulfills all the desirable requirements for a filling material, in addition to there being no consensus in the literature about the best material to be used in the endodontics of deciduous teeth [18–20].

Among the filling materials for deciduous teeth, we have the Guedes Pinto paste and the CTZ antibiotic paste. Guedes-Pinto paste (GP) is composed of iodoform, camphorated paramonochlorophenol and an association of corticoid and antibiotic (Rifocort) and has been widely used due to its antimicrobial and antiseptic properties, in addition to being radiopaque and resorbable, thus, it does not harm the rhizolysis process of the deciduous tooth and the eruption of the permanent successor [21–24]. The CTZ antibiotic paste, on the other hand, is composed of chloramphenicol, tetracycline and eugenol zinc oxide, and has been especially indicated in public health services and in cases of patients who are not cooperative. It presents easy manipulation and biological compatibility; however, there are still controversies regarding the safety of using chloramphenicol [25–28].

The success of endodontic treatment is directly related to intracanal bacterial decontamination and there is a difficulty in endodontic treatment in deciduous teeth, often due to difficult child control, internal anatomy of root canals, and root resorption. Considering these factors, it becomes necessary to know the effectiveness of non-instrumented endodontic treatment (NIET) in deciduous teeth associated with the use of two filling pastes. Therefore, carrying out this clinical study to evaluate the effectiveness of non-instrumental endodontic treatment in deciduous teeth, comparing the performance of CTZ paste and Guedes-Pinto paste, will support the analysis for choosing the most appropriate protocol.

## Materials and methods

### Study design

**Fig 1:**
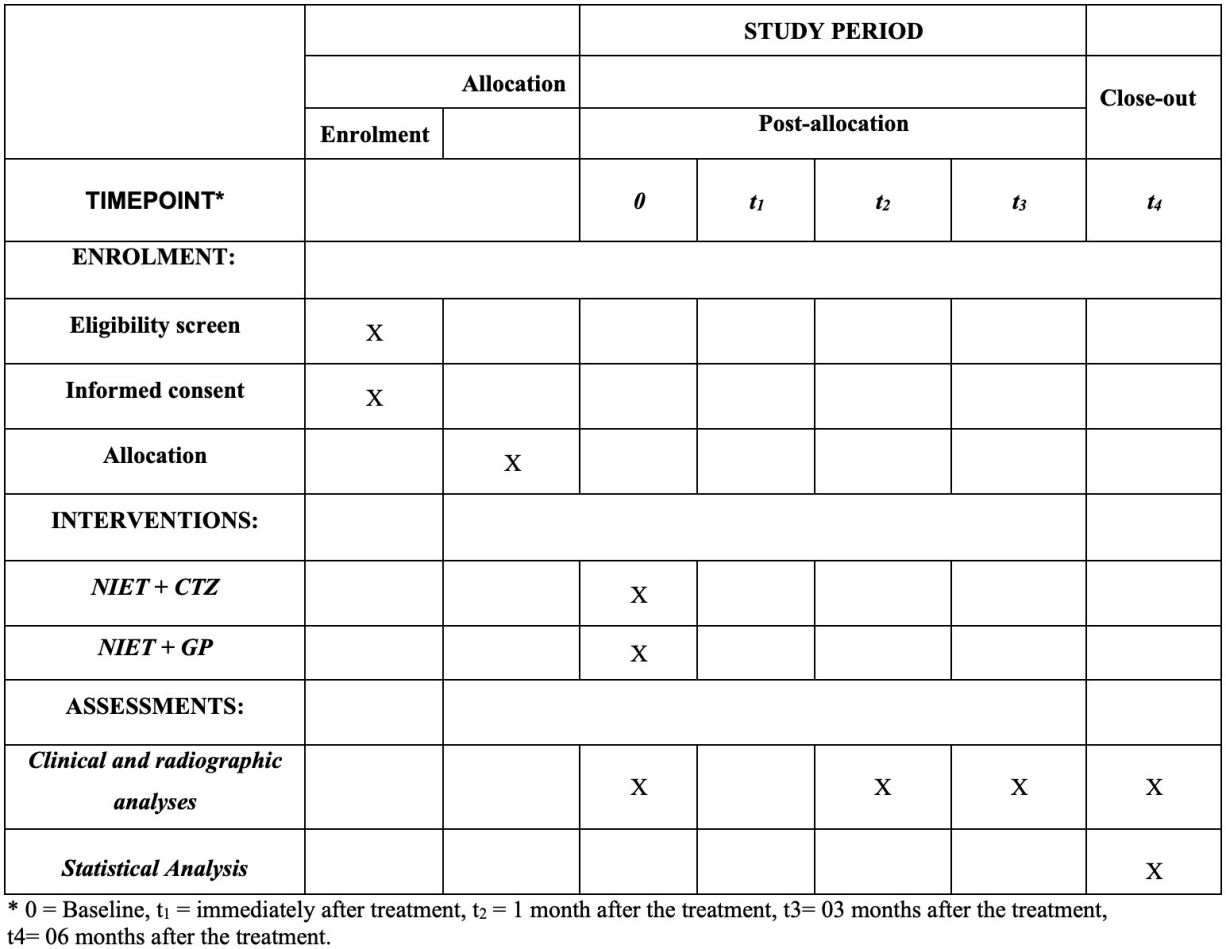
SPIRIT figure as recommended by 2013 SPIRIT statement.

This study protocol will be conducted at the dental clinic of *Universidade Metropolitana de Santos* (UNIMES) and was designed as a as a controlled and randomized clinical trial of non-inferiority, with two parallel arms and allocation rate of 1:1, according to the 2013 Standard Protocol Items: Recommendations for Interventional Trials (SPIRIT) Statement (Fig 1) and the SPIRIT checklist can be found as an additional file.

At the first appointment, a form addressing the medical history of the patient will be completed. Next, the volunteers will be submitted to a clinical examination for the determination of their oral health status. Based on the information collected during this first appointment, the inclusion and exclusion criteria will be applied.

Patients will be selected at the Dental Clinic of *Universidade Metropolitana de Santos* (UNIMES). Recruitment will take place from August 20, 2023 to December 20, 2023. In the first appointment, anamnesis and clinical examinations will be performed to verify if the patient meets the illusion criteria. Patients who meet the inclusion criteria and whose parents or guardians sign the informed consent form will be scheduled for a second appointment for treatment according to the allocation group. The patient should return for the third, fourth and fifth appointment, respectively 1, 3 and 6 months after treatment for clinical and radiographic evaluations.

### Inclusion criteria

Children three to six years of age with at least one anterior primary tooth with irreversible pulpitis or pulp necrosis due to caries or injury with at least 2/3 of the root remaining and not having been submitted to antibiotic therapy in the previous three months.

#### Exclusion criteria

Children with systemic diseases, primary teeth with resorption of 2/3 or more of the root and crypt involvement. Teeth with subgingival lesions, without cervical walls or with unviable restorations after clinical evaluation will be excluded.

### Ethics and dissemination

The study will be conducted in accordance with the ethical precepts stipulated in the Declaration of Helsinki (World Medical Association Declaration of Helsinki, 2008). The protocol for this study was approved by the Human Research Ethics Committee of Universidade Metropolitana de Santos—UNIMES (certificate number: 69464623.2.0000.5509 / 6.054.622—Approval date: May 11, 2023). All information will be contained in the statement of informed consent in accordance with Resolution 196 of the National Board of Health (Health Ministry, Federal District, Brazil, March 10, 1996). The guardians of the children will agree to participate by signing a statement of informed consent; two copies will be signed—one legal guardian of the child and one for the researchers.

The participants will be informed that they may withdraw from the study at any time for any reason if they so desire. The researchers may also remove participants from the study, if deemed necessary.

### Sample calculation

To carry out the sample calculation, a 12-month success rate for the CTZ paste of 86.4% [28] was assumed. The non-inferiority limit was considered as 15%, power of 80% and significance level of 5%, which resulted in 38 teeth per group. 20% were added to this number due to possible sample losses and 40% due to the patient having more than one tooth included, resulting in a sample of 60 deciduous anterior teeth per group, totaling 120 deciduous anterior teeth.

### Randomization of groups

The type of treatment will be randomly determined for each tooth, through a draw before the intervention. The draw will follow the order electronically generated by the randomizer.org randomization website for a balanced distribution of all teeth between the groups.

One hundred and twenty necrotic deciduous teeth of children aged between 3 and 6 years will be selected; and the teeth will be divided into two groups.

Group 1 (G1): NIET + CTZ
Group 2 (G2): NIET + GP

### Interventions

The procedures in all groups will be performed in absolute isolation, but when it’s not possible because the considerable loss of remaining tooth, relative isolation with cotton rolls will be performed.

**Group 1. NIET + CTZ paste**. In group 1, the following endodontic treatment protocol will be performed:

1. Initial X-ray;
2. Anesthetic technique and isolation of the operative field;
3. Removal of tissues with low or high speed burs and/or dentin spoon until exposing the pulp chamber;
4. Removal of the roof of the pulp chamber with inactive tip drills and Of pulp debris. Wash the pulp chamber with saline solution;
5. Locate the root canals;
6. Do the final cleaning with solution;
7. Prepare the CTZ paste: The powder that composes CTZ paste will be previously mixed at a proportion of 1:1:2 (500 mg of chloramphenicol, 500 mg of tetracycline and 1000 mg of zinc oxide) (Fórmula & Ação, São Paulo, Brazil) and incorporated to the eugenol liquid at the time of use with the aid of a flexible nº 24 spatula on a sterile glass plate;
8. Insert the CTZ paste and apply light pressure with cotton balls;
9. Protect the CTZ paste with a thin layer of gutta-percha. Place the slightly warmed gutta-percha on the floor of the pulp chamber, at the entrances of the root canals;
10. Clean the cavity with cotton balls and alcohol;
11. Perform the restoration;
12. Finally, take the final radiograph.

**Group 2. NIET + Guedes-Pinto paste**. In group 2, the following endodontic treatment protocol will be performed:

1. Initial X-ray
2. Perform the anesthetic technique and isolate the operative field;
3. Remove tissues with low or high speed burs and/or dentin spoon until exposing the pulp chamber;
4. Remove the roof of the pulp chamber with inactive tip drills and remove pulp debris. Wash the pulp chamber with saline solution;
5. Locate the root canals;
6. Perform a final saline cleaning of the coronary chamber and dry with sterile cotton balls;
7. Prepare Guedes-Pinto paste by placing 1 cm of Rifocort^®^, 1 cm of iodoform and 2 drops of camphor paramonochlorophenol. Incorporated the substances at the time of use with the aid of a flexible nº 24 spatula on a sterile glass plate;
8. Insert the Guedes-Pinto paste and apply light pressure with cotton balls;
9. Protect the Guedes-Pinto paste with a thin layer of gutta-percha. Place the slightly warmed gutta-percha on the floor of the pulp chamber, at the entrances of the root canals;
10. Clean the cavity with cotton balls and alcohol;
11. Perform the restoration;
12. Finally, perform the final radiograph

### Outcome variables

Clinical evaluations will be considered as the primary outcome and will be performed in a dental chair under the light of a reflector, using a clinical oral mirror and palpation of the affected tooth area. At the initial examination and at the 1, 3 and 6 month follow-up examinations, the following clinical data will be recorded: history of spontaneous pain indicative of apical periodontitis, presence of fistula or abscess, presence of gingival edema, and pathological mobility. As a secondary outcome, signs of radiolucency in the periapical region and pathological root resorption will be evaluated radiographically.

#### Clinical and radiographic analyses of selected teeth

Clinical analyses will be performed in a dental chair under the light of the reflector using a mouth mirror and palpation of the area of the affected tooth. During the initial examination and the follow-up examinations one, three and six months after treatment, the following clinical data will be recorded: history of spontaneous pain indicative of apical periodontitis, presence of fistula or abscess, presence of gingival edema and pathological mobility. The radiographs will be analyzed by two experienced and trained professionals, with the aid of a negatoscope. These professionals will not have any information regarding the treatment group to which each tooth belongs and, in case of doubts during the evaluation, a consensus between the examiners will be established. Radiographically, signs of radiolucency in the periapical region and pathological root resorption will be investigated. Clinical data collected during the initial examination and the follow-up examinations one, three and six months after treatment and the comparison of the initial radiograph for diagnosis and the radiographs taken in the follow-up sessions will serve as the basis for the determination of the success or failure of treatment.

The following criteria based on Chan et al. [10] will be used to determine the success or failure of the proposed treatment (Table 1):

**Table 1:** Criteria for the determination of the success or failure of treatment.

1. Complete repair (= success)	Clinically: absence of signs and symptoms. Radiographically: absence of pathological root resorption, normal width of space of periodontal ligament, absence of development of lesion in periapical region in cases of absence of lesion in initial diagnostic radiograph and complete regression of lesion in cases with presence of lesion prior to treatment.
2. Incomplete repair (= success)	Clinically: absence of signs and symptoms. Radiographically: absence of pathological root resorption and reduction in size of lesion in periapical region.
3. Absence of repair (= failure)	Clinically: signs and symptoms indicative of apical periodontitis in acute phase. Radiographically: presence of pathological root resorption, lesion in furcation/periapical region with unaltered size during follow-up period, increase or development of new radiographic lesion.

All radiographs will be standardized using adult periapical film (Kodak, Rochester, USA) in the occlusal position (modified occlusal radiography). The same development time, intermediate washing, fixation and final washing at all evaluation times will be used to standardized the radiographic process.

### Statistical analysis

The collected data will be tabulated (MS-Office, Excel 2010) and submitted to statistical analysis using the SPSS statistical package (IBM Statistical Package for Social Sciences Version 20; IBM Corporation), in computers to which only the authors will have access and only they will be able to edit the information, mantaining data accuracy and validation. For the primary outcome, the tooth will be the unit of analysis and the Kaplan-Meier test will be performed to estimate the survival rates of the included teeth. In addition, we performed an intention-to-treat (ITT) analysis, considering follow-up successes and failures. For comparison between the two groups, Student’s t test or Mann-Whitney test will be performed, depending on the normality of the data. In addition, Poisson regression analyzes will be carried out, in order to allow the evaluation of the influence of some variables, such as age and sex, in the count of failures in each group. For all analyses, the significance value will be adjusted to 5%.

## Discussion

The aim of this study is to perform a controlled and randomized clinical trial to evaluate the effectiveness of non-instrumental endodontic treatment (NIET) in deciduous teeth with antibiotic paste (CTZ) compared with the efficacy of a iodoform paste (Guedes-Pinto). As limitations of the study, we have a sample that will only represent the Brazilian population and the authors suggest future trials with larger sample sizes and that cover other populations and thus, we will be able to verify the credibility of the results of this study in relation to other samples and populations.

## Supporting information

S1 File SPIRIT checklist. (DOCX)

S2 File Study protocol in original language. (PDF)

S3 File Study protocol in English. (PDF)

S4 File Statement consent in original language. (PDF)

S5 File Statement consent in English. (PDF)
