# Supplementary material for: Efficacy of antibiotic and iodoform pastes in non-instrumental endodontic treatment of anterior primary teeth—Protocol for a randomized controlled clinical
Source: PLoS One. 2023 Sep 8;18(9):e0291133. doi: 10.1371/journal.pone.0291133 (PMC10490878; doi:10.1371/journal.pone.0291133)
Supplement: S2 File — (PDF) [file pone.0291133.s002.pdf]

Eficácia da Pasta Guedes-Pinto e da Pasta CTZ no tratamento endodôntico não instrumentado de dentes decíduos - protocolo de estudo para ensaio clínico controlado e randomizado

Pesquisadora Responsável: Profa. Dra. Ana Paula Taboada Sobral

Santos

2023

Resumo

A manutenção do dente decíduo até que ocorra sua esfoliação fisiológica é um dos principais objetivos da odontopediatria. O tratamento endodôntico em dentes decíduos decorrente de lesões cariosas ou traumáticas com envolvimento pulpar muitas vezes é necessário e frequentemente encontramos uma certa dificuldade em realizá-lo, devido ao difícil controle da criança, a anatomia interna dos canais radiculares, e as reabsorções radiculares. A técnica de tratamento endodôntico não instrumentado (TENI) associado a drogas antimicrobianas, apresenta vantagens como menor tempo de cadeira e menor complexidade que a técnica convencional em que se realizada a instrumentação dos canais radiculares. O objetivo deste estudo é realizar um ensaio clínico controlado e randomizado para comparar a eficácia do tratamento endodôntico não instrumentado (TENI) em dentes decíduos associado a utilização de duas pastas obturadoras. Serão selecionados 120 dentes decíduos necróticos de crianças com idade entre 3 e 6 anos; e os dentes serão divididos em dois grupos. No Grupo1 e no Grupo 2 os canais radiculares não serão instrumentados, apenas irrigados e obturados com as respectivas pastas, CTZ e Guedes-Pinto. Os aspectos radiográficos serão avaliados, considerando o processo de reparação, clinicamente serão avaliados: presença de fístula e mobilidade, as avaliações serão realizadas em ambos os grupos no dia do tratamento e nos períodos de 1, 3 e 6 meses após o tratamento. Para o desfecho principal, o dente será unidade de análise e será realizado o teste de Kaplan-Meier para estimativa das taxas de sobrevida dos dentes incluídos. Para comparação entre os dois grupos, será realizado o teste t de Student ou teste de Mann-Whitney, dependendo da normalidade dos dados. Além disso, análises de regressão de Poisson serão realizadas, de modo a possibilitar a avaliação da influência de algumas variáveis nos resultados. Para todas as análises, o valor de significância será ajustado em 5%.

Palavras-Chave: Tratamento endodôntico, Dentes decíduos, Pasta Guedes-Pinto, necrose pulpar, TENI, CTZ.

1. Introdução

As pesquisas apontam para uma diminuição da cárie dentária na dentição decídua em todo o mundo, apesar disso, a prevalência de cárie continua alta em alguns grupos populacionais. A cárie da infância ainda é muito frequente, pode causar destruição severa e atingir rapidamente a polpa dentária.[1]

As principais causas de inflamação e necrose pulpar em dentes decíduos são as lesões cariosas e traumáticas.[2] A ocorrência de cárie na dentição decídua bastante significante, sendo que cerca de 75% dos dentes com cárie profunda apresentam comprometimento pulpar.[3] Uma vez estabelecida condição de irreversibilidade da inflamação pulpar, ou a necrose tecidual, o tratamento endodôntico radical deve ser realizado.[2] Existem 2 opções de tratamento para dentes decíduos com polpa infectada e ou necrótica: extração ou pulpectomia.[1]

A pulpectomia consiste na remoção completa de células necróticas e da polpa irreversivelmente infectada de um dente afetado, para que o dente permaneça assintomático e funcional na cavidade oral até esfoliar normalmente.[4] A perda prematura dos dentes decíduos pode produzir mudanças no guia de erupção do dente permanente, o que pode levar a distúrbios fonéticos e hábitos orais prejudiciais, como a interposição da língua e as consequências estéticas.[5-8]

A terapia pulpar em dentes decíduos é um tratamento complexo, principalmente pelos procedimentos de instrumentação, complexidade do delta apical, ciclo biológico dos dentes decíduos, reabsorção fisiológica radicular e rizólise e longas sessões de tratamento durante as quais a criança às vezes nem sempre coopera.[5,6,9]

A técnica mais utilizada para o tratamento endodôntico em dentes decíduos é realizada com limas manuais e soluções irrigadoras desinfectantes. A instrumentação mecânica associada à irrigação química não elimina totalmente os micro-organismos presentes no canal radicular.[10-16] Assim, o objetivo do tratamento endodôntico é a máxima desinfecção do sistema de canais rediculares (SCR)[12,17,-19] e a prevenção da reinfecção.

A técnica denominada terapia de esterilização da lesão e reparo tecidual (Lesion sterilization and tissue repair therapy - LSTR) foi proposta para dentes decíduos não vitais, com avançada reabsorção radicular, dentes estrategicamente importantes, com perda óssea, mobilidade, radiolucência na área de furca, pacientes não cooperativos e que não podem se submeter a extração naquele momento. Esta técnica, contudo, está

contraindicada para pacientes alérgicos a algum dos componentes dos agentes antibióticos utilizados, extensa reabsorção interna ou externa, dentes próximos a exfoliação, perfuração do assoalho pulpar e crianças com endocardite bacteriana.[20] A combinação de medicamentos é utilizada para minimizar a quantidade de microrganismos presentes na lesão ou no canal radicular e espera-se o reparo dos tecidos se a lesão é desinfectada.[21,22] Quando aplicada para tratamento de polpas necrosadas em dentes decíduos, é realizada sem a instrumentação dos canais radiculares, ou seja, sem a preparação químico-mecânica convencional e uma associação de antimicrobianos é depositada nas entradas dos canais radiculares. A abordagem LSTR ou tratamento endodôntico não instrumental (TENI) envolve, portanto, o uso de uma associação de drogas antimicrobianas e apresenta vantagens como menor tempo, menor complexidade, prevenção da irritação dos tecidos periapicais e do germe do permanente sucessor, além de poder ser utilizada em dentes decíduos que apresentem processo de rizólise de até mais de um terço das raízes.[23-24]

Os materiais obturadores de dentes decíduos devem apresentar as seguintes propriedades: reabsorvível, radiopaco, bactericida, promover adequado preenchimento e aderência às paredes dos canais radiculares, facilmente removido quando necessário; além de não provocar danos aos tecidos periapicais e ao germe do dente permanente, e tampouco alteração da coloração das estruturas dentárias. Porém, não existe um único material que preencha todos os requisitos desejáveis para um material obturador, além de não haver consenso na literatura sobre o melhor material a ser utilizado na endodontia de dentes decíduos.[25-27]

Dentre os materiais obturadores de dentes decíduos temos a pasta Guedes Pinto e a pasta antibiótica CTZ. A pasta Guedes-Pinto é composta por iodofórmio, paramonoclorofenol canforado e uma associação de corticoide e antibiótico (Rifocort) tem sido bastante utilizada, devido às suas propriedades antimicrobianas e anti sépticas, além de ser radiopaca e reabsorvível, assim, não prejudica o processo de rizólise do dente decíduo e o irrompimento do permanente sucessor.[28] Já a pasta antibiótica de CTZ é composta por cloranfenicol, tetraciclina e óxido de zinco eugenol tem sido especialmente indicada em serviços públicos de saúde e em casos de pacientes que não sejam colaborativos. Apresenta fácil manipulação, compatibilidade biológica; no entanto, ainda existem controvérsias quanto à segurança no emprego do cloranfenicol. [29,30]

O sucesso do tratamento endodôntico está diretamente relacionado com a descontaminação bacteriana intracanal e existe uma dificuldade do tratamento

endodôntico em dentes decíduos, muitas vezes pelo difícil controle da criança, anatomia interna dos canais radiculares, e reabsorções radiculares. Considerando esses fatores, se torna necessário conhecer a eficácia do tratamento endodôntico não instrumentado (TENI) em dentes decíduos associado a utilização de duas pastas obturadoras. Portanto, realizar este estudo clínico para avaliar a efetividade do tratamento endodôntico não instrumental em dentes decíduos comparando o desempenho da pasta CTZ e da Pasta Guedes-Pinto, irá subsidiar a análise para a escolha do protocolo mais adequado.

2. MÉTODOS

2.1 Objetivo Geral

O objetivo deste estudo é realizar um ensaio clínico controlado e randomizado para avaliar a eficácia do tratamento endodôntico não instrumental (TENI) em dentes decíduos com a pasta CTZ (pasta a base de antibióticos) comparada com a eficácia da Pasta Guedes- Pinto (pasta a base de iodofórmio).

2.2 Delineamento Experimental

O presente trabalho se caracteriza como estudo ensaio clínico controlado e randomizado de não inferioridade, com dois braços paralelos e taxa de alocação de 1:1 e que será realizado nas dependências da Clínica Odontológica da Universidade Metropolitana de Santos (UNIMES). Por se tratar de um estudo clínico randomizado e buscando uma maior transparência e qualidade dessa pesquisa, utilizaremos as recomendações CONSORT (Consolidated Standards of ReportingTrials). O protocolo do estudo foi registrado na plataforma internacional de ensaios clínicos Clinical Trials, com número de registro NCT04587089

2.3 Aspectos Éticos

O estudo será conduzido eticamente de acordo com os critérios descritos na Declaração de Helsinki (World Medical Association Declaration of Helsinki, 2008). O protocolo desse estudo será submetido à aprovação do Comitê de Ética em Pesquisa da Universidade Metropolitana de Santos (UNIMES). Todas as informações estarão presentes no Termo de consentimento livre e esclarecido (Resolução no. 196 do Conselho

Nacional de Saúde, Ministério da Saúde, Distrito Federal, Brasil, 10/03/1996), os quais serão assinados em duas vias, pertencendo uma ao responsável, e outra aos pesquisadores.

Os participantes do estudo receberão também instruções de que poderão desistir do estudo a qualquer momento, por qualquer razão, se assim o desejarem. Os pesquisadores também poderão remover os participantes do estudo, caso achem necessário.

2.4 Determinação do tamanho da amostra

Para a realização do cálculo amostral, assumiu-se uma taxa de sucesso em 12 meses para a pasta CTZ de 86,4%[31]. Considerou-se como 15% o limite de não inferioridade, poder de 80% e nível de significância de 5% o que resultou em 38 dentes por grupo. Adicionou-se a esse número 20% devido a possíveis perdas amostrais e 40% devido ao paciente poder ter mais de um dente incluído, resultando em uma amostra de 60 dentes anteriores decíduos por grupo, totalizando 120 dentes anteriores decíduos.

2.5 População do estudo

Na primeira consulta um formulário contendo a história médica do paciente também será preenchido. Na sequência, esses voluntários serão submetidos a exame clínico, para a determinação das suas condições orais. Com base nas informações coletadas nessa primeira visita, o estudo seguirá os critérios de Inclusão e Exclusão.

2.5.1 Critérios de inclusão

Crianças com idade de 3 a 6 anos, com pelo menos um dente decíduo anterior com pulpite irreversível ou necrose pulpar por cárie ou trauma, com pelo menos 2/3 de raiz remanescente e crianças que não tenham sido submetidas á terapia com antibiótico nos três meses anteriores.

2.5.2 Critérios de exclusão

Crianças com a saúde comprometida, com dentes decíduos com reabsorção de 2/3 ou mais de raiz, e envolvimento de cripta.

2.6 Grupos de Estudo

GRUPO	INTERVENÇÃO
**G1**	TENI + CTZ
**G2**	TENI + Pasta Guedes-Pinto

2.7 Randomização

O tipo de tratamento será determinado aleatoriamente para cada dente, através da realização de um sorteio antes da intervenção. O sorteio seguirá ordem gerada eletronicamente pelo site de randomização *randomizer.org* para distribuição de maneira equilibrada de todos os dentes entre os grupos.

2.8 Intervenções

2.8.1 Grupo 1. TENI + Pasta CTZ

No grupo 1 será realizado o seguinte protocolo de tratamento endodôntico:

1. Radiografia inicial
2. Realizar a técnica anestésica e isolar o campo operatório;
3. Remover os tecidos com brocas de baixa ou alta rotação e/ou colher de dentina até expor a câmara pulpar;
4. Realizar a remoção do teto da câmara pulpar com brocas de ponta inativa e remover restos pulpares. Lavar a câmara pulpar com solução salina;
5. Localizar os canais radiculares;
6. Fazer a limpeza final com solução salina da câmara coronária e secar com bolinhas de algodão estéreis;
7. Preparar a pasta CTZ: O pó que compõe a pasta CTZ será manipulado na proporção 1:1:2 (500mg de Cloranfenicol, 500mg de Tetraciclina e 1.000mg de Óxido de Zinco) pela Fórmula & Ação (F&A) e incorporado ao líquido eugenol no momento de sua utilização, com o auxílio de uma espátula nº 24 flexível e sobre uma placa de vidro estéril.do pó da pasta CTZ juntamente com eugenol;
8. Inserir a pasta CTZ e com bolinhas de algodão fazer leve pressão;

9. Fazer a proteção da pasta CTZ com uma camada fina de guta-percha. Colocar a guta-percha levemente aquecida e acondicioná-la no assoalho da câmara pulpar, nas entradas dos canais radiculares cuidadosamente;
10. Limpar a cavidade com bolinhas de algodão e álcool;
11. Realizar a restauração;
12. Por fim, realizar a radiografia final

2.8.2 Grupo 2. TENI + Pasta Guedes-Pinto

No grupo 2 será realizado o seguinte protocolo de tratamento endodôntico:

1. Radiografia inicial
2. Realizar a técnica anestésica e isolar o campo operatório;
3. Remover os tecidos com brocas de baixa ou alta rotação e/ou colher de dentina até expor a câmara pulpar;
4. Realizar a remoção do teto da câmara pulpar com brocas de ponta inativa e remover restos pulpares. Lavar a câmara pulpar com solução salina;
5. Localizar os canais radiculares;
6. Fazer a limpeza final com solução salina da câmara coronária e secar com bolinhas de algodão estéreis;
7. Preparar a pasta Guedes-Pinto: Colocar 1 cm Rifocort®, 1 cm de Iodofórmio e 2 gotas de Paramonoclorofenol Canforado (PMCC), incorporar os medicamentos com o auxílio de uma espátula nº 24 flexível e sobre uma placa de vidro estéril.
8. Inserir a pasta Guedes-Pinto e com bolinhas de algodão fazer leve pressão;
9. Fazer a proteção da pasta Guedes-Pinto com uma camada fina de guta-percha. Colocar a guta-percha levemente aquecida e acondicioná-la no assoalho da câmara pulpar, nas entradas dos canais radiculares cuidadosamente;
10. Limpar a cavidade com bolinhas de algodão e álcool;
11. Realizar a restauração;
12. Por fim, realizar a radiografia final

2.9 Avaliações Clínicas e radiográficas dos dentes selecionados

As avaliações clínicas serão consideradas como desfecho primário e serão realizadas em cadeira odontológica sob a luz do refletor, usando espelho clínico bucal e palpação da área do dente afetado. No exame inicial e nos exames de controle de 1, 3 e

6 meses, os seguintes dados clínicos serão registrados: história de dor espontânea indicativa de periodontite apical, presença de fístula ou abcesso, presença de edema gengival, e mobilidade patológica. Como desfecho secundário, radiograficamente serão avaliados sinais de radiolucência na região periapical e de reabsorção radicular patológica.

Os dados clínicos coletados no exame inicial e no controle de 1, 3 e 6 meses após o tratamento e a comparação da radiografia inicial para diagnóstico com a radiografias realizadas nos exames de controle serão base para avaliação do sucesso ou insucesso da terapêutica endodôntica. As radiografias serão analisadas por dois profissionais experientes e treinados, com o auxílio de negatoscópio. Estes profissionais não terão nenhuma informação com relação ao grupo de tratamento ao qual cada dente pertencia e, em caso de dúvidas durante a avaliação será estabelecido um consenso entre os examinadores.

Os seguintes critérios serão utilizados para a determinação de sucesso ou insucesso do tratamento proposto, segundo o trabalho de Chan et al [15]:

1.Reparo completo (= sucesso)	Clinicamente: ausência de sinais e sintomas.<br>Radiograficamente: ausência de reabsorção radicular patológica, largura do espaço do ligamento periodontal normal, ausência de desenvolvimento de lesão na região de periapical nos casos de ausência de lesão observada na radiografia inicial para diagnóstico e regressão total da lesão quando presente no início do tratamento.
2. Reparo incompleto (= sucesso)	Clinicamente: ausência de sinais e sintomas.<br>Radiograficamente: ausência de reabsorção radicular patológica e redução em tamanho da lesão na região de periapical.
3.Ausência de reparo (= insucesso)	Clinicamente: sinais e sintomas indicativos de periodontite apical em fase aguda.<br>Radiograficamente: presença de reabsorção radicular patológica, lesão na região de furca/periapical de tamanho inalterado durante o período de acompanhamento, aumento ou desenvolvimento de nova lesão radiográfica.

Todas as radiografias serão padronizadas, usando filme periapical adulto (Kodak, Rochester, NY, EUA), na posição oclusal (radiografia oclusal modificada).

O mesmo tempo de revelação, lavagem intermediária, fixação e a lavagem final em todos os tempos de avaliação serão usados para padronizar o processamento da radiografia.

2.10 Análise estatística

Para o desfecho primário, o dente será unidade de análise e será realizado o teste de Kaplan-Meier para estimativa das taxas de sobrevida dos dentes incluídos. Além disso, realizamos uma análise de intenção de tratar (ITT), considerando o sucesso e as falhas no acompanhamento. Para comparação entre os dois grupos, será realizado o teste t de Student ou teste de Mann-Whitney, dependendo da normalidade dos dados. Além disso, análises de regressão de Poisson serão realizadas, de modo a possibilitar a avaliação da influência de algumas variáveis nos resultados. Para todas as análises, o valor de significância será ajustado em 5%.

3.Resultados Esperados

Por meio do presente trabalho poderemos avaliar, se haverá diferença de efetividade entre os tratamentos propostos, bem como se Pasta Guedes-Pinto na técnica TENI apresentará resultados clínicos e radiográficos iguais ou melhores quando comparados com a pasta CTZ.

.

4. Cronograma de Execução

**Atividades**	Pré intervenção			1	2	3	4	5	6	7	8	9	10
Submissão Comitê de Ética em Pesquisa	■	■											
Seleção dos participantes e randomização			■										
Procedimentos				■	■	■	■	■	■	■	■		
Coleta dos dados				■	■	■	■	■	■	■	■		
Análise dos dados											■	■	■
Redação e apresentação dos resultados obtidos												■	■
Submissão dos artigos científicos													■
Relatório final													■

5. Referencias

1. SARI S, OKTE Z. Success rate of sealapex in root canal treatment for primary teeth: 3-year follow-up. Oral Surg Med Pathol Oral Radiol Endod 2008; 105; e93-96.
2. MASSARA MLA, TAVARES WLF, NORONHA JC, HENRIQUE LCF, RIBEIRO Sobrinho AP, A Eficácia do Hidróxido de Cálcio no Tratamento Endodôntico de Decíduos: Seis Anos de Avaliação. Pesq Bras Odontoped Clin. Integr, 2012 ;abr/jun 12(2):155-59.
3. COSER RM, GIRO EMA. Tratamento endodôntico de molares decíduos humanos com necrose pulpar e lesão periapical. PGR- Pós-Grad Rev Fac Odontol São José dos Campos.2002 jan/abr; 5(1):84-92.
4. NAVIT S, JAISWAL N, KHAN SA, MALHOTRA S, SHARMA A, MUKISH, JABEEN S, AGARWAL G. Antimicrobial Efficacy of Contemporary Obturating Materials used in Primary Teeth- An In-vitro Study. Jour of Clin and Diag Res. 2016 Sep; 10(9): 9-12.
5. FABRIS AS, NAKANO V, AVILA-CAMPOS MJ. Bacteriological analysis of necrotic pulp and fistulae in primary teeth. 2014; 22(2);118-124.
6. PINHEIRO SL, ARAUJO G, BINCELLI I, CUNHA R, BUENO C. Evaluation of cleaning capacity and instrumentation time of manual, hybrid and rotary instrumentation techniques in primary molars. Inter Endod Jour. 2012 Apr; 45(4):379-385.
7. KOSHY S, LOVE RM. Endodontic Treatment In The Primary Dentition. Australian Endo Jour. 2004;30(2):59-68.
8. AMARAL RR, SÁ DM, MENEZES AJAC, Terapia Fotodinâmica Antimicrobiana na Endodontia: revisão de literatura. Pós em Rev do Centro Univ Newton Paiva. 2015; (11): 39-41.
9. FERREIRa FV, ANGONESE MP, FRIEDRICH HC, WEISS RDN, FRIEDRICH RS, PRAETZEL JR. Antimicrobial Action of root canal filing pastes used in deciduous teeth. Rev odonto ciênc. 2010; 25(1):65-68.
10. TRICHES TC, FIGUEIREDO LC, FERES M, FREITAS SFT, ZIMMERMANN GS, CORDEIRO MMR. Microbial Reduction by Two Chemical-Mechanical Protocols in Primary Teeth with Pulp Necrosis and Periradicular Lesion – An In Vivo Study. Brazilian Dental Journal. 2014; 25(4): 307 – 313.

11. SOUZA LC, BRITO PRR, OLIVEIRA JCM, ALVES FRFA, MOREIRA EJL et al. Photodynamic Therapy with Two Different Photosensitizers as a Supplement to Instrumentation/Irrigation Procedures in Promoting Intracanal Reduction of Enterococcus faecalis. JOE 2010 Febr;36(2):292-296.

12. RIOS A, HE J, GLICKMAN GN, SPEARS R, SCHNEIDERMAN ED, HONEYMAN AI. Evaluation of photodynamic therapy using a light-emitting diode lamp against Enterococcus faecalis in extractic human teeth. Joe 2011 jun; 37(6):856-859.

13. TRINDADE AC, FIGUEIREDO JAP, STEIER L, WEBER JBB. Photodynamic Therapy in Endodontics: A Literature Review.Photomed and Laser Surg. 2015; 33(3):175-182.

14. GARCEZ AS, ROQUE JA, MURATA WH, HAMBLIN MN. Uma nova estratégia para PDT antimicrobiana em Endodontia. Rev Assoc Cir Dent .2016;70(2): 126-130.

15. CHAN EKM, DESMEULES M, CIELECKI M, DABBAGH B, SANTOS BF. Longitudinal Cohort Study of Regenerative Endodontic Treatment for Immature Necrotic Permanent Teeth. Journal of Endodontics 2017 March; 43(3):395-400.

16. SILVA FC, FREITAS LRP, LOURENÇO APA, BRAGA JUNIOR ACR, JORGE AOC, OLIVEIRA LD. Analysis of the effectiveness of the instrumentation associated to antimicrobial photodynamic therapy and root canal dressing in the elimination of Enterococcus faecalis in root canals. Braz DentSci 2010 jan/jun; 13 (5) 31-38.

17. NEENA IE, ANANTHRAJ A, PRAVEEN P, KARTHIK V, RANI P. Comparison of digital radiography and apex locator with the conventional method in root length determination of primary teeth. Jour of Indian Society of Pedodont and Preven Dentis.2011 Oct-Dec; 29(4): 300-3004.

18. CHREPA V, KOTSAKIS GA, PAGONIS TC, HARGREAVES KM. The Effect of Photodynamic Therapy in Root Canal Disinfection: A Systematic Review. 2014 July; 40(7): 891-895.

19. FIMPLE JL, FONTANA CR, FOSCHI F, RUGGIERO K, SONG X, PAGONIS TC, TANNER ACR, KENT R et al. Photodynamic treatment of endodontic polymicrobial infection in vitro. J Endod. 2008 June; 34(6): 728-734.

20. SAIN, S.; RESHMI, L.; ANANDARAJ, S.; SAGEENA, G. et al. Lesion Sterilization and Tissue Repair-Current Concepts and Practices. Int J Clin Pediatr Dent., v. 11, n. 55, p. 446-450, 2018.

21. Takushire T, Cruz EV, Aasgormoarl A, Hoshino E. Endodontic treatment of primary teeth using a combination of antibacterial drugs. Int Endodon J 2004; 37(2):132-8.

22. Hoshino E, Kurihara-Ando N, Sato I, Uematsu H, Sato M, Kota K, Iwaku M. In-vitro antibacterial susceptibility of bacteria taken from infected root dentine to a mixture of ciprofloxacin, metronidazole and minocycline. Int Endod J 1996 Mar;29(2):125-30. doi: 10.1111/j.1365-2591.1996.tb01173.x. PMID: 9206436.

23. Capiello J. Tratamentos pulpares em incisivos primários. Rev Assoc Odontol Argentina 1964;52:139-45,

24. Capiello J. Nuevos enfoques em odontologia infantil. Odontol Uruguai 1967; 23:23-30.

25. FUKS, A. B. ; EIDELMAN, E. Pulp therapy in the primary dentition. Current Opinion in Dentistry. 1991, Vol. 1, 556-563.

26. MORTAZAVI, M. e MESBAHI, M. Comparison of zinc oxide and eugenol, and Vitapex for root canal treatment of necrotic primary teeth. International Journal of Paediatric Dentistry. 2004, Vol. 14,6,.417-424.

27. PINTO, D. N.; SOUSA, D. L.; ROCHA, R. B. et al. Eighteen-month clinical and radiographic evaluation of two root canal-filling materials in primary teeth with pulp necrosis secondary to trauma. Dental Traumatology. 2011, Vol. 27, 3, pp. 221-224.

28. MASSARA, Maria de Lourdes Andrade et al. A eficácia do hidróxido de cálcio no tratamento endodôntico de decíduos: seis anos de avaliação. Pesquisa Brasileira em Odontopediatria e Clínica Integrada, v. 12, n. 2, p. 155-159, 2012.

29. Barros, E. V. R. & Neres, S. D. A. N. Terapia pulpar em dentes decíduos utilizando pasta de CTZ composta de Clorafenicol, Tetraciclina e Óxido de Zinco e Eugenol: uma revisão de literatura.(2017).(Trabalho de Conclusão de Curso apresentado na Faculdade Integrada de Pernambuco para obtenção de título de bacharel em Odontologia). Faculdade Integrada de Pernambuco.

30. LINDOSO, T. K. N. .; CARVALHO, W. C. .; THOMES, C. R. .; NÓBREGA, D. L. S. da .; TEIXEIRA, R. D. P. .; LIMA , Élida C. da S. .; MELO, W. . B. de .; SOUZA, M. M. F. .; BARROS, M. . M. .; OLIVEIRA, I. F. F. de .; ANTUNES, A. A. .; MARQUES, W. R. .; PEREIRA, A. F. A. .; LOBATO , L. S. .; FARIAS , T. C. .;

CANTANHEDE , L. M. . The employability of CTZ paste in the endodontic treatment of primary dentition: a literature review. Research, Society and Development, [S. l.], v. 10, n. 17, p. e226101724696, 2021. DOI: 10.33448/rsd-v10i17.24696. Disponível em: https://rsdjournal.org/index.php/rsd/article/view/24696. Acesso em: 30 mar. 2022.

31. Moura J, Lima M, Nogueira N, Castro M, Lima C, Moura M, Moura L. LSTR Antibiotic Paste Versus Zinc Oxide and Eugenol Pulpectomy for the Treatment of Primary Molars with Pulp Necrosis: A Randomized Controlled Trial. Pediatr Dent. 2021 Nov 15;43(6):435-442. PMID: 34937613.
