# Supplementary material for: Efficacy of antibiotic and iodoform pastes in non-instrumental endodontic treatment of anterior primary teeth—Protocol for a randomized controlled clinical
Source: PLoS One. 2023 Sep 8;18(9):e0291133. doi: 10.1371/journal.pone.0291133 (PMC10490878; doi:10.1371/journal.pone.0291133)
Supplement: S3 File — (PDF) [file pone.0291133.s003.pdf]

Efficacy of Guedes-Pinto Paste and CTZ Paste in non-instrumented endodontic treatment of primary teeth - study protocol for a randomized controlled clinical trial

Responsible Researcher: Prof. Dr. Ana Paula Taboada Sobral

Santos

2023

Summary

The maintenance of the deciduous tooth until its physiological exfoliation occurs is one of the main objectives of pediatric dentistry. Endodontic treatment in deciduous teeth resulting from carious or traumatic lesions with pulpal involvement is often necessary and we often find it difficult to perform it, due to the difficult control of the child, the internal anatomy of the root canals, and root resorptions. The non-instrumented endodontic treatment technique (NIET) associated with antimicrobial drugs has advantages such as shorter chair time and less complexity than the conventional technique in which root canal instrumentation is performed. The aim of this study is to carry out a controlled and randomized clinical trial to compare the effectiveness of non-instrumented endodontic treatment (NIET) in primary teeth associated with the use of two obturator pastes. 120 necrotic deciduous teeth of children aged between 3 and 6 years will be selected; and the teeth will be divided into two groups. In Group 1 and Group 2, the root canals will not be instrumented, just irrigated and filled with the respective pastes, CTZ and Guedes-Pinto. The radiographic aspects will be evaluated, considering the repair process, clinically will be evaluated: presence of fistula and mobility, the evaluations will be carried out in both groups on the day of treatment and in periods of 1, 3 and 6 months after treatment. For the main outcome, the tooth will be the unit of analysis and the Kaplan-Meier test will be performed to estimate the survival rates of the included teeth. For comparison between the two groups, Student's t test or Mann-Whitney test will be performed, depending on the normality of the data. In addition, Poisson regression analyzes will be carried out, in order to allow the evaluation of the influence of some variables on the results. For all analyses, the significance value will be adjusted to 5%.

Key words: Endodontic treatment, Primary teeth, Guedes-Pinto paste, pulp necrosis, NIET, CTZ.

1. Introduction

Research points to a decrease in dental caries in the primary dentition worldwide, despite this, the prevalence of caries remains high in some population groups. Childhood caries is still very common, it can cause severe destruction and quickly reach the dental pulp.1

The main causes of inflammation and pulpal necrosis in deciduous teeth are carious and traumatic injuries.2 The occurrence of caries in the deciduous dentition is quite significant, with approximately 75% of teeth with deep caries having pulp involvement.3 Once the condition of pulp is established, irreversibility of pulpal inflammation, or tissue necrosis, radical endodontic treatment should be performed.2 There are 2 treatment options for deciduous teeth with infected and/or necrotic pulp: extraction or pulpectomy.1

Pulpectomy consists of the complete removal of necrotic cells and irreversibly infected pulp from an affected tooth, so that the tooth remains asymptomatic and functional in the oral cavity until it exfoliates normally.4 Premature loss of deciduous teeth can produce changes in the tooth eruption guide permanent, which can lead to phonetic disorders and harmful oral habits, such as tongue interposition and aesthetic consequences.5-8

Pulp therapy in deciduous teeth is a complex treatment, mainly due to instrumentation procedures, complexity of the apical delta, biological cycle of deciduous teeth, physiological root resorption and rhizolysis, and long treatment sessions during which the child sometimes does not always cooperate.5 ,6,9

The most used technique for endodontic treatment in deciduous teeth is performed with manual files and disinfectant irrigating solutions. Mechanical instrumentation associated with chemical irrigation does not completely eliminate microorganisms present in the root canal.10-16 Thus, the objective of endodontic treatment is maximum disinfection of the root canal system (RCS)12,17,-19 and prevention of reinfection.

The technique called Lesion sterilization and tissue repair therapy (LSTR) was proposed for non-vital deciduous teeth, with advanced root resorption, strategically important teeth, with bone loss, mobility, radiolucency in the furcation area, uncooperative patients who cannot undergo extraction at that time. This technique, however, is contraindicated for patients allergic to any of the components of the antibiotic agents used, extensive internal or external resorption, teeth close to exfoliation, perforation of the pulp floor and children with bacterial endocarditis.20 The combination

of drugs is used to minimize the amount of microorganisms present in the lesion or in the root canal and tissue repair is expected if the lesion is disinfected.21, 22 When applied to treat necrotic pulps in deciduous teeth, it is performed without instrumenting the root canals, that is, without conventional chemical-mechanical preparation and an association of antimicrobials is deposited in the entrances of the root canals. The LSTR approach or non-instrumental endodontic treatment (NIET) therefore involves the use of an association of antimicrobial drugs and has advantages such as less time, less complexity, prevention of irritation of the periapical tissues and the germ of the permanent successor, in addition to being able to be used in deciduous teeth that present a rhizolysis process of up to more than a third of the roots.23-24 without the conventional chemical-mechanical preparation and an association of antimicrobials is deposited in the entrances of the root canals. The LSTR approach or non-instrumental endodontic treatment (NIET) therefore involves the use of an association of antimicrobial drugs and has advantages such as less time, less complexity, prevention of irritation of the periapical tissues and the germ of the permanent successor, in addition to being able to be used in deciduous teeth that present a rhizolysis process of up to more than a third of the roots.23-24 without the conventional chemical-mechanical preparation and an association of antimicrobials is deposited in the entrances of the root canals. The LSTR approach or non-instrumental endodontic treatment (NIET) therefore involves the use of an association of antimicrobial drugs and has advantages such as less time, less complexity, prevention of irritation of the periapical tissues and the germ of the permanent successor, in addition to being able to be used in deciduous teeth that present a rhizolysis process of up to more than a third of the roots.23-24

The filling materials for deciduous teeth must have the following properties: resorbable, radiopaque, bactericidal, promote adequate filling and adherence to the walls of the root canals, easily removed when necessary; in addition to not causing damage to the periapical tissues and to the permanent tooth germ, nor altering the color of dental structures. However, there is no single material that fulfills all the desirable requirements for a filling material, in addition to there being no consensus in the literature about the best material to be used in the endodontics of deciduous teeth.25-27

Among the filling materials for deciduous teeth, we have the Guedes Pinto paste and the CTZ antibiotic paste. The Guedes-Pinto paste is composed of iodoform, camphorated paramonochlorophenol and an association of corticoid and antibiotic (Rifocort) and has been widely used due to its antimicrobial and antiseptic properties, in addition to being

radiopaque and resorbable, thus, it does not harm the process of rhizolysis of the deciduous tooth and the eruption of the permanent successor.28 The CTZ antibiotic paste, on the other hand, is composed of chloramphenicol, tetracycline and eugenol zinc oxide has been especially indicated in public health services and in cases of patients who are not cooperative. It presents easy manipulation, biological compatibility; however, there are still controversies regarding the safety of using chloramphenicol. 29.30

The success of endodontic treatment is directly related to intracanal bacterial decontamination and there is a difficulty in endodontic treatment in deciduous teeth, often due to difficult child control, internal anatomy of root canals, and root resorption. Considering these factors, it becomes necessary to know the effectiveness of non-instrumented endodontic treatment (NIET) in deciduous teeth associated with the use of two filling pastes. Therefore, carrying out this clinical study to evaluate the effectiveness of non-instrumental endodontic treatment in deciduous teeth, comparing the performance of CTZ paste and Guedes-Pinto paste, will support the analysis for choosing the most appropriate protocol.

2. METHODS

2.1 Main goal

The aim of this study is to carry out a controlled and randomized clinical trial to evaluate the effectiveness of endodontic treatmentnon-instrumental (NIET) in deciduous teeth with CTZ paste (antibiotic-based paste) compared with the effectiveness of Guedes-Pinto Paste (iodoform-based paste).

2.2 Experimental Design

The present work is characterized as a controlled and randomized clinical trial.of non-inferiority, with two parallel arms and allocation rate of 1:1 and which will be carried out at the Dental Clinic of the Metropolitan University of Santos (UNIMES). Because it is a randomized clinical trial and seeking greater transparency and quality of this research, we will use the CONSORT recommendations (Consolidated Standards of ReportingTrials). The study protocol was registered on the international platform of clinical trials Clinical Trials, with registration number NCT04587089

2.3 Ethical aspects

The study will be conducted ethically according to the criteria described in the Declaration of Helsinki (World Medical Association Declaration of Helsinki, 2008). The protocol for this study will be submitted for approval by the Research Ethics Committee of the Metropolitan University of Santos (UNIMES). All information will be present in the free and informed consent form (Resolution no. 196 of the National Health Council, Ministry of Health, Federal District, Brazil, 03/10/1996), which will be signed in two copies, one belonging to the responsible, and another to the researchers.

Study participants will also be instructed that they may withdraw from the study at any time, for any reason, if they so choose. Researchers will also be able to remove participants from the study if they deem it necessary.

2.4 Determination of sample size

To carry out the sample calculation, a 12-month success rate for the CTZ paste of 86.4%31 was assumed. The non-inferiority limit was considered as 15%, power of 80% and significance level of 5%, which resulted in 38 teeth per group. 20% were added to this number due to possible sample losses and 40% due to the patient having more than one tooth included, resulting in a sample of 60 deciduous anterior teeth per group, totaling 120 deciduous anterior teeth.

2.5 Study population

At the first consultation, a form containing the patient's medical history will also be completed. Subsequently, these volunteers will undergo a clinical examination to determine their oral conditions. Based on the information collected in this first visit, the study will follow the Inclusion and Exclusion criteria.

2.5.1 Inclusion criteria

Children aged 3 to 6 years, with at least one anterior deciduous tooth with irreversible pulpitis or pulp necrosis due to caries or trauma, with at least 2/3 of root remaining, and children who have not undergone antibiotic therapy within three months previous.

2.5.2 Exclusion criteria

Children with compromised health, with deciduous teeth with 2/3 or more root resorption, and crypt involvement.

2.6 Study Groups

GROUP	INTERVENTION
G1	NIET + CTZ
G2	NIET + Guedes-Pinto Paste

2.7 Randomization

The type of treatment will be randomly determined for each tooth, through a draw before the intervention. The draw will follow the order electronically generated by the randomizer.org randomization website for a balanced distribution of all teeth between the groups.

2.8 Interventions

2.8.1 <u>Group 1. NIET + CTZ Paste</u>

In group 1, the following endodontic treatment protocol will be performed:

1. Initial X-ray
2. Perform the anesthetic technique and isolate the operative field;
3. Remove tissues with low or high speed burs and/or dentin spoon until exposing the pulp chamber;
4. Remove the roof of the pulp chamber with inactive tip drills and remove pulp debris. Wash the pulp chamber with saline solution;
5. Locate the root canals;
6. Perform a final saline cleaning of the coronary chamber and dry with sterile cotton balls;
7. Prepare the CTZ paste: The powder that makes up the CTZ paste will be manipulated in a 1:1:2 ratio (500mg of Chloramphenicol, 500mg of Tetracycline and 1,000mg of Zinc Oxide) by Formula & Action (F&A) and incorporated into

the liquid eugenol at the time of use, with the aid of a flexible spatula nº 24 and on a sterile glass plate.

8. Insert the CTZ paste and apply light pressure with cotton balls;

9. Protect the CTZ paste with a thin layer of gutta-percha. Place the slightly warmed gutta-percha and place it on the floor of the pulp chamber, carefully at the entrances of the root canals;

10. Clean the cavity with cotton balls and alcohol;

11. Perform the restoration;

12. Finally, perform the final radiograph

2.8.2 Group 2. NIET + Guedes-Pinto Paste

In group 2, the following endodontic treatment protocol will be performed:

1. Initial X-ray
2. Perform the anesthetic technique and isolate the operative field;
3. Remove tissues with low or high speed burs and/or dentin spoon until exposing the pulp chamber;
4. Remove the roof of the pulp chamber with inactive tip drills and remove pulp debris. Wash the pulp chamber with saline solution;
5. Locate the root canals;
6. Perform a final saline cleaning of the coronary chamber and dry with sterile cotton balls;
7. Prepare the Guedes-Pinto paste: Place 1 cm of Rifocort®, 1 cm of Iodoform and 2 drops of Camphorated Paramonochlorophenol (PMCC), incorporate the medicines with the help of a flexible spatula nº 24 and on a sterile glass plate.
8. Insert the Guedes-Pinto paste and apply light pressure with cotton balls;
9. Protect the Guedes-Pinto paste with a thin layer of gutta-percha. Place the slightly warmed gutta-percha and place it on the floor of the pulp chamber, carefully at the entrances of the root canals;
10. Clean the cavity with cotton balls and alcohol;
11. Perform the restoration;
12. Finally, perform the final radiograph

2.9 Clinical and radiographic evaluations of selected teeth

Clinical evaluations will be considered as the primary outcome and will be performed in a dental chair under the light of a reflector, using a clinical oral mirror and palpation of the affected tooth area. At the initial examination and at the 1, 3 and 6 month follow-up examinations, the following clinical data will be recorded: history of spontaneous pain indicative of apical periodontitis, presence of fistula or abscess, presence of gingival edema, and pathological mobility. As a secondary outcome, signs of radiolucency in the periapical region and pathological root resorption will be evaluated radiographically.

The clinical data collected in the initial examination and in the control of 1, 3 and 6 months after the treatment and the comparison of the initial radiograph for diagnosis with the radiographs taken in the control exams will be the basis for evaluating the success or failure of the endodontic therapy. The radiographs will be analyzed by two experienced and trained professionals, with the aid of a negatoscope. These professionals will not have any information regarding the treatment group to which each tooth belonged and, in case of doubts during the evaluation, a consensus between the examiners will be established.

The following criteria will be used to determine the success or failure of the proposed treatment, according to the work by Chan et al 15:

1.Complete repair (=success)	Clinically: absence of signs and symptoms.<br>radiographically: absence of pathological root resorption, normal width of the periodontal ligament space, absence of lesion development in the periapical region in cases of absence of lesion observed in the initial radiograph for diagnosis and total regression of the lesion when present at the beginning of treatment.
2. Incomplete repair (= success)	Clinically: absence of signs and symptoms.<br>radiographically: absence of pathological root resorption and reduction in size of the lesion in the periapical region.
3. Failure to repair (= failure)	Clinically: signs and symptoms indicative of apical periodontitis in the acute phase.<br>radiographically: presence of pathological root resorption, lesion in the furcation/periapical region of unchanged size during the follow-up period, enlargement or development of a new radiographic lesion.

All radiographs will be standardized using adult periapical film (Kodak, Rochester, NY, USA) in the occlusal position (modified occlusal radiograph).

The same development time, intermediate wash, fixation and final wash at all evaluation times will be used to standardize radiography processing.

2.10 Statistical analysis

For the primary outcome, the tooth will be the unit of analysis and the Kaplan-Meier test will be performed to estimate the survival rates of the included teeth. In addition, we performed an intention-to-treat (ITT) analysis, considering follow-up successes and failures. For comparison between the two groups, Student's t test or Mann-Whitney test will be performed, depending on the normality of the data. In addition, Poisson regression analyzes will be carried out, in order to allow the evaluation of the influence of some variables on the results. For all analyses, the significance value will be adjusted to 5%.

3. Expected Results

Through the present work, we will be able to assess whether there will be a difference in effectiveness between the proposed treatments, as well as whether Guedes-Pinto paste in the NIET technique will present equal or better clinical and radiographic results when compared to the CTZ paste.

4. Execution Schedule

**Activities**	Pre intervention			1	two	3	4	5	6	7	8	9	10
Submission Research Ethics Committee													
Selection of participants and randomization													
Procedures													
data collection													
Data analysis													
Writing and presenting the results obtained													
Submission of scientific articles													
Final report													

5. References

1. SARI S, OKTE Z. Success rate ofsealapex in root canal treatment for primary teeth: 3-year follow-up. Oral Surg Med Pathol Oral Radiol Endod 2008; 105; e93-96.

two. MASSARA MLA, TAVARES WLF, NORONHA JC, HENRIQUE LCF, RIBEIRO Sobrinho AP, The Efficacy of Calcium Hydroxide in Primary Endodontic Treatment: Six Years of Evaluation.Pesq Bras Odontoped Clin. Integr, 2012 ;Apr/Jun 12(2):155-59.

3. COSER RM, GIRO EMA. Endodontic treatment of human primary molars with pulp necrosis and periapical lesion. PGR- Post-Grad Rev Fac Odontol São José dos Campos.2002 Jan/Apr; 5(1):84-92.

4. NAVIT S, JAISWAL N, KHAN SA, MALHOTRA S, SHARMA A, MUKISH, JABEEN S, AGARWAL G. Antimicrobial Efficacy of Contemporary Obturating Materials used in Primary Teeth- An In-vitro Study. Journey of Clin andDiag Res. 2016 Sep; 10(9): 9-12.

5. FABRIS AS, NAKANO V, AVILA-CAMPOS MJ.Bacteriological analysis of necrotic pulp and fistulae in primary teeth. 2014; 22(2);118-124.

6. PINHEIRO SL, ARAUJO G, BINCELLI I, CUNHA R, BUENO C. Evaluation of cleaning capacity and instrumentation time of manual,hybrid and rotary instrumentation techniques in primary molars. Inter Endod Jour. 2012 Apr; 45(4):379-385.

7. KOSHY S, LOVE RM. Endodontic TreatmentIn The Primary Dentition. Australian Endo Jour. 2004;30(2):59-68.

8. AMARAL RR, SÁ DM, MENEZES AJAC, Antimicrobial Photodynamic Therapy in Endodontics: literature review. Post inRev do Centro Univ Newton Paiva. 2015; (11): 39-41.

9. FERREIRA FV, ANGONESE MP, FRIEDRICH HC, WEISS RDN, FRIEDRICH RS, PRAETZEL JR. Antimicrobial Action of root canal filing pastes used in deciduous teeth. Rev odonto ciênc. 2010; 25(1):65-68.

10. TRICHES TC, FIGUEIREDO LC, FERES M, FREITAS SFT, ZIMMERMANN GS, CORDEIRO MMR.Microbial Reduction by Two Chemical-Mechanical Protocols in Primary Teeth with Pulp Necrosis and Periradicular Lesion – An In Vivo Study. Brazilian Dental Journal. 2014; 25(4): 307 – 313.

11. SOUZA LC, BRITO PRR, OLIVEIRA JCM, ALVES FRFA, MOREIRA EJL et al.Photodynamic Therapy with Two Different Photosensitizers as a Supplement to Instrumentation/Irrigation Procedures in Promoting Intracanal Reduction of Enterococcus faecalis. JOE 2010 Febr;36(2):292-296.

12. RIOS A, HE J, GLICKMAN GN, SPEARS R, SCHNEIDERMAN ED, HONEYMAN AI. Evaluation of photodynamic therapy using a light-emitting diode lamp against Enterococcus faecalis inextractic human teeth. Joe 2011 Jun; 37(6):856-859.

13. TRINDADE AC, FIGUEIREDO JAP, STEIER L, WEBER JBB.Photodynamic Therapy in Endodontics: A Literature Review. Photomed and Laser Surg. 2015; 33(3):175-182.

14. GARCEZ AS, ROQUE JA, MURATA WH, HAMBLIN MN. A new strategy for antimicrobial PDT in Endodontics.Rev Assoc Cir Dent .2016;70(2): 126-130.

15. CHAN EKM, DESMEULES M, CIELECKI M, DABBAGH B, SANTOS BF. Longitudinal Cohort Study of Regenerative Endodontic Treatment for Immature Necrotic Permanent Teeth.Journal of Endodontics 2017 March; 43(3):395-400.

16. SILVA FC, FREITAS LRP, LOURENÇO APA, BRAGA JUNIOR ACR, JORGE AOC, OLIVEIRA LD.Analysis of the effectiveness of the instrumentation associated with antimicrobial photodynamic therapy and root canal dressing in the elimination of Enterococcus faecalis in root canals. Braz DentSci 2010 Jan/Jun; 13 (5) 31-38.

17. NEENA IE,ANANTHRAJ A, PRAVEEN P, KARTHIK V, RANI P. Comparison of digital radiography and apex locator with the conventional method in root length determination of primary teeth. Jour of Indian Society of Pedodont and Preven Dentis.2011 Oct-Dec; 29(4): 300-3004.

18. CHREPA V, KOTSAKIS GA, PAGONIS TC, HARGREAVES KM. The Effect of Photodynamic Therapy in Root Canal Disinfection: A Systematic Review. 2014 July; 40(7): 891-895.

19. FIMPLE JL, FONTANA CR, FOSCHI F, RUGGIERO K, SONG X, PAGONIS TC, TANNER ACR, KENT R et al.Photodynamic treatment of endodontic polymicrobial infection in vitro. J Endod. 2008 June; 34(6): 728-734.

20. SAIN, S.; RESHMI, L.; ANANDARAJ, S.; SAGEENA, G. et al.Lesion Sterilization and Tissue Repair-Current Concepts and Practices. Int J Clin Pediatr Dent., v. 11, no. 55, p. 446-450, 2018.

21. Takushire T, Cruz EV, Aasgormoarl A, Hoshino E. Endodontic treatment of primary teeth using a combination of antibacterial drugs. Int Endodon J 2004; 37(2):132-8.
22. Hoshino E,Kurihara-Ando N, Sato I, Uematsu H, Sato M, Kota K, Iwaku M. In-vitro antibacterial susceptibility of bacteria taken from infected root dentine to a mixture of ciprofloxacin, metronidazole and minocycline. Int Endod J 1996 Mar;29(2):125-30. doi: 10.1111/j.1365-2591.1996.tb01173.x. PMID: 9206436.
23. Capiello J. Pulp treatments in primary incisors. Rev Assoc Odontol Argentina 1964;52:139-45,
24. Capiello J. New approaches in pediatric dentistry. Odontol Uruguay 1967; 23:23-30.
25. FUKS, A.B. ; EIDELMAN, E. Pulp therapy in the primary dentition. Current Opinion in Dentistry. 1991, Vol. 1, 556-563.
26. MORTAZAVI, M. and MESBAHI, M. Comparison of zinc oxide and eugenol, andVitapex for root canal treatment of necrotic primary teeth. International Journal of Pediatric Dentistry. 2004, Vol. 14,6, 417-424.
27. PINTO, DN; SOUSA, DL; ROCHA, RB et al. Eighteen-month clinical and radiographic evaluation of two root canal-filling materials in primary teeth with pulp necrosis secondary to trauma.Dental Traumatology. 2011, Vol. 27, 3, pp. 221-224.
28. MASSARA, Maria de Lourdes Andrade et al. The effectiveness of calcium hydroxide in primary endodontic treatment: six years of evaluation. Brazilian Research in Pediatric Dentistry and Integrated Clinic, v. 12, no. 2, p. 155-159, 2012.
29. Barros, EVR & Neres, SDAN Pulp therapy in deciduous teeth using CTZ paste composed ofChloramphenicol, Tetracycline and Zinc Oxide and Eugenol: a literature review.(2017). Integrated Faculty of Pernambuco.
30. BEAUTIFUL, TKN. .; CARVALHO, WC .; THOMES, CR.; NÓBREGA, DLS da .; TEIXEIRA, RDP .; LIMA , Élida C. da S. .; MELO, W. . B. of.; SOUZA, MMF .; BARROS, M. . M. .; OLIVEIRA, IFF de .; ANTUNES, AA .; MARQUES, WR.; PEREIRA, AFA .; LOBATO, LS.; FARIAS, TC.; CANTANHEDE, LM. The employability of CTZ paste in the endodontic treatment of primary dentition: a literature review. Research, Society and Development, [S. l.], v. 10, no. 17, p. e226101724696, 2021. DOI: 10.33448/rsd-v10i17.24696. Available at: https://rsdjournal.org/index.php/rsd/article/view/24696. Accessed on: 30 Mar. 2022.

31. Moura J, Lima M, Nogueira N, Castro M, Lima C, Moura M, Moura L. LSTR Antibiotic Paste Versus Zinc Oxide and Eugenol Pulpectomy for the Treatment of Primary Molars with Pulp Necrosis: A Randomized Controlled Trial.Pediatr Dent. 2021 Nov 15;43(6):435-442. PMID: 34937613.
