# Supplementary material for: Efficacy of antibiotic and iodoform pastes in non-instrumental endodontic treatment of anterior primary teeth—Protocol for a randomized controlled clinical
Source: PLoS One. 2023 Sep 8;18(9):e0291133. doi: 10.1371/journal.pone.0291133 (PMC10490878; doi:10.1371/journal.pone.0291133)
Supplement: S4 File — (PDF) [file pone.0291133.s004.pdf]


TERMO DE CONSENTIMENTO LIVRE E ESCLARECIDO

Eficácia da Pasta Guedes-Pinto e da Pasta CTZ no tratamento endodôntico não instrumentado de dentes decíduos - protocolo de estudo para ensaio clínico controlado e randomizado

Número do CAAE:

Você está sendo convidado a participar como voluntário de uma pesquisa cujo título é **Eficácia da Pasta Guedes-Pinto e da Pasta CTZ no tratamento endodôntico não instrumentado de dentes decíduos - protocolo de estudo para ensaio clínico controlado e randomizado.** Este documento, chamado Termo de Consentimento Livre e Esclarecido, visa assegurar seus direitos como participante e é elaborado em duas vias, uma que deverá ficar com você e outra com o pesquisador.

Por favor, leia com atenção e calma. Se houver perguntas antes ou mesmo depois de assiná-lo, você poderá esclarecê-las com o pesquisador. Não haverá nenhum tipo de penalização ou prejuízo se você não aceitar participar ou retirar sua autorização em qualquer momento.

Justificativa e objetivos:

Justificativa: Tendo em vista que o sucesso do do tratamento endodôntico está diretamente relacionado com a descontaminação bacteriana intracanal e existe uma dificuldade do tratamento endodôntico em dentes decíduos, muitas vezes pelo difícil controle da criança, anatomia interna dos canais radiculares, e reabsorções radiculares. Considerando esses fatores, se torna necessário conhecer a eficácia do tratamento endodôntico não instrumentado (TENI) em dentes decíduos associado a utilização de duas pastas obturadoras.

Objetivos: O objetivo deste estudo é realizar um ensaio clínico controlado e randomizado para avaliar a eficácia do tratamento endodôntico não instrumental (TENI) em dentes decíduos com a pasta CTZ (pasta a base de antibióticos) comparada com a eficácia da Pasta Guedes- Pinto (pasta a base de iodofórmio).

Procedimentos:

A pesquisa será realizada com pacientes de ambos os sexos matriculados regularmente na Clínica de Odontologia da Universidade Metropolitana de Santos (UNIMES).

O tipo de tratamento será determinado aleatoriamente para cada dente, através da realização de um sorteio antes da intervenção.

Grupo 1. Sem Instrumentação + Pasta CTZ - Neste grupo os canais não serão instrumentados e a obturação será realizada com a pasta CTZ. Proteção da pasta com uma fina camada de guta-percha e restauração.

RUBRICA DO PARTICIPANTE DA PESQUISA

RUBRICA DO PESQUISADOR

TERMO DE CONSENTIMENTO LIVRE E ESCLARECIDO – (TCLE)

Grupo 2. Sem Instrumentação + Pasta Guedes-Pinto - Neste grupo os canais não serão instrumentados e a obturação será realizada com a Pasta Guedes- Pinto. Proteção da pasta com uma fina camada de guta-percha e restauração.

Desconfortos e riscos:

O paciente poderá apresentar dor ou desconforto no procedimento anestésico e após tratamento, sendo algumas vezes necessária a utilização de analgésicos nos dias seguintes.

Benefícios:

Os voluntários e seus responsáveis participarão das atividades de educação em saúde bucal com aconselhamento de alimentação e higiene. Os voluntários terão a boca examinada e se houver necessidade serão encaminhadas para tratamento odontológico.

Acompanhamento e assistência:

A qualquer momento, antes, durante ou até o término da pesquisa, nos colocamos a disposição para o esclarecimento de qualquer dúvida sobre a pesquisa.

Sigilo e privacidade:

Você tem a garantia de que sua identidade será mantida em sigilo. Os dados coletados serão utilizados exclusivamente para fins da pesquisa, e que poderão ser apresentados em eventos de natureza científica e/ou publicados em revistas, sem revelar a identidadedos participantes.

Ressarcimento e Indenização:

Caso esta pesquisa cause, comprovadamente, qualquer custo ou dano procure o pesquisador responsável a fim de ressarcimento ou possível indenização.

Contato:

Em caso de dúvidas sobre a pesquisa, se precisar consultar esse registro de consentimento ou quaisquer outras questões, você poderá entrar em contato com os pesquisadores:

Nome do pesquisador responsável:
Endereço: Ana Paula Taboada Sobral
E-mail: anapaula@taboada.com.br

Nome do discente pesquisador
Endereço:
Telefone:
E-mail:

RUBRICA DO PARTICIPANTE DA PESQUISA

RUBRICA DO PESQUISADOR

TERMO DE CONSENTIMENTO LIVRE E ESCLARECIDO – (TCLE)

Em caso de denúncias ou reclamações sobre sua participação e sobre questões éticas do estudo, você poderá entrar em contato com a secretaria do Comitê de Ética em Pesquisa da Universidade Metropolitana de Santos (das 08h30 às 11h30 e das 13h00 às 17h) na Avenida Conselheiro Nébias, 536 - 2. andar. Santos- SP. E-mail: cpq@unimes.br

Consentimento Livre e Esclarecido:
Após ter recebido esclarecimentos sobre a natureza da pesquisa, seus objetivos, procedimentos, benefícios previstos, potenciais riscos e o incômodo que este estudo pode acarretar, aceito participar:

Nome do(a) participante: ____

____ Data: ____/_____/______.
(Assinatura do participante ou nome e assinatura do seu RESPONSÁVEL LEGAL)

Responsabilidade do Pesquisador:
Asseguro ter explicado e fornecido uma via deste documento ao participante. Informo que o estudo foi aprovado pelo CEP perante o qual o projeto foi apresentado. Comprometo-me a utilizar o material e os dados obtidos nesta pesquisa exclusivamente para as finalidades previstas neste documento ou conforme o consentimento dado pelo participante. Os dados serão apresentados em congressos e publicados em revistas, apenas sem a identificação dos participantes.

_____________________________________. Data: ____/_____/______.
(Assinatura do pesquisador)
