# Supplementary material for: Efficacy of antibiotic and iodoform pastes in non-instrumental endodontic treatment of anterior primary teeth—Protocol for a randomized controlled clinical
Source: PLoS One. 2023 Sep 8;18(9):e0291133. doi: 10.1371/journal.pone.0291133 (PMC10490878; doi:10.1371/journal.pone.0291133)
Supplement: S5 File — (PDF) [file pone.0291133.s005.pdf]


STATEMENT CONSET

Efficacy of Guedes-Pinto Paste and CTZ Paste in non-instrumented endodontic treatment of primary teeth - study protocol for a randomized controlled clinical trial
Number of CAAE:

You are being invited to participate as a volunteer in a survey whose title is **Efficacy of Guedes-Pinto Paste and CTZ Paste in non-instrumented endodontic treatment of deciduous teeth - study protocol for a randomized controlled clinical trial.** This document, called the Term of Free and Informed Consent, aims to ensure your rights as a participant and is prepared in two ways, one that should stay with you, and another with the researcher.

Please, read with attention and calm. If there are questions before after signing it, you can clarify them with the researcher. There will be no type of penalty or prejudice if you no to accept to participate or to remove your authorization in anytime.

Justification It is goals:
Justification: Considering that the success of endodontic treatment is directly related to intracanal bacterial decontamination and there is a difficulty in endodontic treatment in deciduous teeth, often due to difficult child control, internal anatomy of root canals, and root resorption. Considering these factors, it becomes necessary to know the effectiveness of non-instrumented endodontic treatment (NIET) in deciduous teeth associated with the use of two filling pastes.

Goals: The aim of this study is to carry out a controlled and randomized clinical trial to evaluate the effectiveness of non-instrumental endodontic treatment (NIET) in deciduous teeth with CTZ paste (antibiotic-based paste) compared with the effectiveness of Guedes-Pinto paste (a paste based on antibiotics). iodoform base).

Procedures:
A search will be carried out with patients of both you sexes enrolled regularly at the Clinic of Dentistry from the Universidade Metropolitana de Santos (UNIMES).

The type of treatment will be randomly determined for each tooth, through the realization of a raffle before from the intervention.

Group 1. No Instrumentation + CTZ Folder - In this group the canals will not be instrumented and obturation will be performed with CTZ paste. Folder protection with a thin layer of gutta percha and restoration.

RUBRICA DO PARTICIPANTE DA PESQUISA

RUBRICA DO PESQUISADOR

Group 2. Without Instrumentation + Guedes-Pinto Paste - In this group, the canals do not will be instrumented and the obturation will be performed with Pasta Guedes-Pinto. Protection of folder with a thin layer in gutta-percha and restoration.

Discomfort and risks:

The patient can present pain or discomfort at the anesthetic procedure or after treatment, being sometimes necessary to use painkillers in the following days.

Benefits:

Volunteers and their guardians will participate in education activities in oral health with advice on nutrition and hygiene. Volunteers will have the mouth examined to check if there is a need for treatment dental.

Follow-up It is assistance:

At any time, before, during or until the end of the research, we are in disposition for clarification of any doubt about the search.

Secrecy It is privacy:

You have the guarantee that your identity will be maintained in secrecy. Data collected will be used exclusively for research. It may be presented in events of scientific nature and/or published in journals, without revealing the identity of the participants.

Refund It is Indemnity:

In case the research causes, demonstrably, any cost or damage, look for the responsible researcherto for reimbursement or possible indemnity.

Contact:

In case of doubts about the search, you can enter in contact with the researchers:

Name of researcher responsible:
Address: Ana Paula Taboada Sobral
Email: anapaula@taboada.com.br

Name of research student
Address:
Telephone:
Email:

RUBRICA DO PARTICIPANTE DA PESQUISA

RUBRICA DO PESQUISADOR

STATEMENT CONSET

In case of denouncements or complaints about your participation and ethical issues of the study, you can contact the secretariat of the Research Ethics Committee of Universidade Metropolitana de Santos (from 08:30 am to the 11:30 am, and 13:00 pm to the 5 pm) at Avenue Counselor Nébias, 536 - Santos- SP. Email: cpq@unimes.br.

Consent Free It is Enlightened:
After receiving clarifications about the nature of the research, its goals, procedures, anticipated benefits, potential risks, and the inconvenience this study may entail, I accept to participate:

Name of participant: ____

_______________________________________ Date: ____/_____/______.
(Signature of participant or responsible party)

Responsibility of Researcher:
I assure that I have explained and provided a copy of this document to the participant. I inform you that the study was approved by the CEP before the project was presented. I promise to use the material and data obtained in this research exclusively for the purposes planned in this document, according the consent given by the participant. Data will be presented in congresses and published in jounals, without participant identification.

____________________________________. Date: ____/_____/______.
(Signature of researcher)
